# Investigating the Efficacy of Adhesive Tape for Drilling Carbon Fibre Reinforced Polymers

**DOI:** 10.3390/ma14071699

**Published:** 2021-03-30

**Authors:** Chander Prakash, Alokesh Pramanik, Animesh K. Basak, Yu Dong, Sujan Debnath, Subramaniam Shankar, Sunpreet Singh, Linda Yongling Wu, Hongyu Y. Zheng

**Affiliations:** 1 School of Mechanical Engineering, Shandong University of Technology, Zibo 255000, Shandong, China; chander.mechengg@gmail.com (C.P.); zhenghongyu@sdut.edu.cn (H.Y.Z.); 2 School of Mechanical Engineering, Lovely Professional University, Phagwara, Punjab 144411, India; 3 School of Civil and Mechanical Engineering, Curtin University, Bentley, WA 6102, Australia; alokesh.pramanik@curtin.edu.au (A.P.); Y.Dong@curtin.edu.au (Y.D.); 4 Adelaide Microscopy, University of Adelaide, Adelaide, SA 5005, Australia; animesh.basak@adelaide.edu.au; 5 Department of Mechanical Engineering, Curtin University Malaysia, Miri 98009, Malaysia; d.sujan@curtin.edu.my; 6 Department of Mechatronics Engineering, Kongu Engineering College, Erode 638060, India; shankariitm@gmail.com; 7 Department of Mechanical Engineering, National University of Singapore, Singapore 119077, Singapore; snprt.singh@gmail.com

**Keywords:** drilling, carbon fibre reinforced polymers (CFRPs), thrust force, surface finish, drilling accuracy, delamination, adhesive tape

## Abstract

In the present research work, an effort has been made to explore the potential of using the adhesive tapes while drilling CFRPs. The input parameters, such as drill bit diameter, point angle, Scotch tape layers, spindle speed, and feed rate have been studied in response to thrust force, torque, circularity, diameter error, surface roughness, and delamination occurring during drilling. It has been found that the increase in point angle increased the delamination, while increase in Scotch tape layers reduced delamination. The surface roughness decreased with the increase in drill diameter and point angle, while it increased with the speed, feed rate, and tape layer. The best low roughness was obtained at 6 mm diameter, 130° point angle, 0.11 mm/rev feed rate, and 2250 rpm speed at three layers of Scotch tape. The circularity error initially increased with drill bit diameter and point angle, but then decreased sharply with further increase in the drill bit diameter. Further, the circularity error has non-linear behavior with the speed, feed rate, and tape layer. Low circularity error has been obtained at 4 mm diameter, 118° point angle, 0.1 mm/rev feed rate, and 2500 RPM speed at three layers of Scotch tape. The low diameter error has been obtained at 6 mm diameter, 130° point angle, 0.12 mm/rev feed rate, and 2500 rpm speed at three layer Scotch tape. From the optical micro-graphs of drilled holes, it has been found that the point angle is one of the most effective process parameters that significantly affects the delamination mechanism, followed by Scotch tape layers as compared to other parameters such as drill bit diameter, spindle speed, and feed rate.

## 1. Introduction

Carbon fibre reinforced polymers (CFRPs) bind carbon fibres and polymer matrices together and possess inorganic and synthetic characteristics. The applications of CFRPs are in demand, nowadays, for structural and functional components due to their high toughness, strength, and stiffness [1]. Unlike many other engineering materials, CFRPs have a wide range of promising applications in automotive and aerospace industries, benefiting from their lightweight structures. The application areas include, but are not limited to, telecommunication, automotive, oil and gas, building and construction, sports and recreation, aviation, biomedical, marine (naval), electronics, defence or military, power generation, consumer products, and food and packaging industries [2]. It is well known that CFRP components are made in the form of panels and complete large structures, and are built by joining different panels by means of mechanical fasteners [1]. However, it is challenging to apply specific processes, particularly drilling, on CFRPs due to the presence of reinforced carbon fibres. The properties of carbon fibres are completely different from those of matrix materials. The fibres cause very high tool wear and delamination at the entry and exit of the drilled holes [3,4]. Various types of drill bits, such as standard twist drills, twist drills with double point angles, drills with multiple flutes, ‘brad & spur’ drills, dagger drills, step drills, core drills, saw drills, step-core drills, and core-special drills have been used for drilling CFRPs [5,6]. Among these, the standard point geometry twist drill is considered the most common type of tool geometry, and is used in most engineering applications. Typical point angle within common drilling process is 118°. The tip of the drill bit initiates the spindle process within the workpiece, which is the most important part of the drill bit as it affects the overall drilling performance [7,8]. Furthermore, overall drilling performance is also affected by machining parameters and flute and flank shapes [7,8].

There are many studies which aid understanding of the drilling process of different fibre reinforced plastics. For example, Davim et al. [9] investigated the effect of cemented carbide drill bits with different geometries, speeds, and feeds on the thrust force while machining glass fibre reinforced plastics. Based on their statistical analysis, it was shown that specific cutting pressure was reduced as the feed and speed increased, though the thrust force was enhanced as the feed increased. The spur drill bit produced smaller thrust force than that of 118° point angle drill bit. The feed had the highest impact, particularly on drilling pressure and thrust force. Davim et al. [10] also investigated the effect of spindle speed, feed, and resin types on specific cutting force, delamination factor, and surface roughness while drilling unsaturated polyester and propoxylated bisphenol A-fumarate based composites using cemented carbide tools. It was reported that unsaturated polyester-based composites had a smaller specific cutting pressure than that of propoxylated bisphenol A-fumarate-based counterparts, where feed rate was found to make significant contributions to both composite systems. Unsaturated polyester-based composites exhibited lowest surface damage while surface roughness increased with feed and speed. Tsao [11] studied the effect of diameter ratio, feed, and speed on thrust force and delamination including drilling CFRP using compound core and core-saw drill bits where core-saw drills yielded better results than core drills. Feed and speed contributed to thrust force and delamination significantly while the effect of diameter ratio was negligible. Tsao and Chiu [12] assessed thrust force while drilling CFRPs with compound core-special and step-core-special drills. It was noted that cutting velocity ratio, feed, and drill types (twist, saw and candlestick types) highly influenced thrust force. A high negative cutting velocity ratio (inner and outer parts spinning in opposite directions) reduced thrust force significantly.

Khashaba [13] reported that the influence of chisel edge on thrust force was in the range of 40 to 60% of total thrust. It was suggested to consider the drill bits with the capability to distribute thrust towards drill periphery rather than concentrate on the centre to generate delamination-free holes. In addition, pre-machining methods, such as step drills, pilot holes, and back support, might reduce the delamination. Reduced feed near hole exit also reduces the delamination. It was also mentioned that high drilling temperatures, low thermal conductivity, and low glass transition temperature of the composites might induce matrix pyrolysis, composite fracture, and accelerated tool wear. Kumar and Singh [14] reported that thrust force and torque in conventional drilling of CFRP were greater than those of rotatory ultrasonic conventional drilling. Thrust force increased with increasing feed rate while modified tool geometry reduced thrust force instead.

Khanna et al. [15] drilled CFRPs under a dry and cryogenic environment, which reported that the average surface roughness of drilled holes decreased by 14–38%, and the entry delamination factor was reduced by 5–68% due to the cryogenic application. While drilling thick CFRPs, Geng et al. [16] noted that higher temperature in the drilling region might induce chip adhesion to newly generated surfaces. The addition of ultrasonic elliptical vibration could diminish the temperature effects on machined surfaces. Feito et al. [17] stated that the point angle of new drill bits had minor effects on thrust force, which, however, was considerable for a worn drill bit while drilling CFRPs. The delamination factors at the exit and entry of drilled holes were enhanced with increasing point angle. Drill bit wear reduced the delamination at entry, which then increased at the exit end. The statistical analysis revealed that tool wear, point angle, and feed most significantly impacted thrust force despite minor contribution from the speed.

Feito et al. [18] also used numerical models to analyse the delamination while drilling CFRP. The drill bit was spinning in one model and became stationary in another model. The stationary drill bit worked as a punch and pierced the workpiece. This arrangement overestimated the delamination factor where the effect of thrust force on delamination factor was investigated. It was concluded that highest amount of delamination reached a plateau at a specific thrust force. The numerical and experimental studies conducted by Phadnis et al. [19] considering complex kinematics at drill-workpiece (CFRP composites) indicated that thrust force, torque, and delamination increased with increasing feed rate, but decreased progressively as the cutting speed increased. It was also reported that lower feed and higher speed gave better outcomes with respect to drilling CFRP. Merino-Pérez et al. [20] found that the types of thermosetting matrix materials significantly affected thrust force and torque, while the types of carbon fibres and cutting speed had little effect on thrust force. Moreover, the speed strongly influenced the torque. As the modulus of CFRPs increased, the sensitivity to speed and strain rate also improved accordingly. The strength and failure characteristics of CFRPs were influenced by the behaviour of matrix materials. The effect of speed on torque was ascribed to the negative effect of strain rate on the ability of the matrix to transfer the load to reinforcements. Ahmad, Khan, and Raza [21] used orbital drilling based on the workpiece rotation, which reported that tool wear, surface roughness, and diametric error were reduced due to less loading taking place on cutting tool. The performance of coated tools was found to be better than their uncoated counterparts on all output responses. The above-mentioned discussion has shown that most investigations are related to the effect of different drilling parameters on surface roughness, delamination, thrust force, and torque in relation to machining CFRPs [22,23]. It has been found that delamination occurs at the exit and entry of the drilling process. Additionally, a supporting material layer might also help to reduce the delamination at the entry stage of the drilling process [24,25].

The applications of carbon fibre reinforced polymers (CFRPs) have recently increased for manufacturing light weight structural/functional components due to their high toughness, strength, and stiffness [26]. Owing to this, CFRPs have dominated the applications of conventional engineering materials in automotive and aerospace industries. However, drilling of CFRPs always encounters a critical challenge, in the assembly lines, due to the presence of carbon fibres. This present study aims to understand the effectiveness of Scotch tape layers at the drill entry to reduce the delamination. There is an urgent need to investigate the effect of Scotch tape layers along with other different input parameters on drilling forces, circularity, diameter error, cylindricity, surface roughness, and delamination of drilled holes. Therefore, this study investigates the effect of speed, feed and tool point angle on circularity, diameter error, and cylindricity of drilled holes in addition to thrust force, torque, and surface roughness. The outcomes of this investigation might be beneficial to machining and tooling using CFRPs by correlating their diverse input and output parameters.

## 2. Materials and Methods

**Figure 1:**
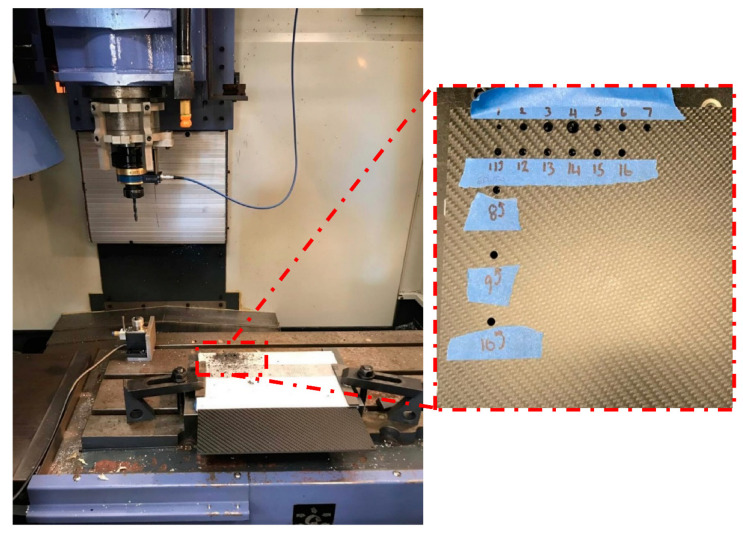
Experimental set-up for drilling of CFRP.

The CFRP sheet (EconomyPlate) with 5 mm thickness and 120 × 120 cm^2^ area was manufactured by DragonPlate and further used in this study. The sheet consists of 0°/90° layer orientation composed entirely of a tough and rigid carbon reinforced epoxy matrix. The resin system is Bisphenol A—Room Temp Cure. The hardener was not disclosed by the supplier. The commonly available Scotch tape with inbuilt adhesive was used for this primary investigation. The properties (thickness 0.18 mm) of the Scotch tape and adhesives are not available. The other details of experiments are given as follows: (a) material of drill bits: HSS Steel, (b) diameters of drill bits: 4, 6, 8 and 10 mm, (c) point angle of drill bits:118, 125, 130 and 140°, (d) number of Scotch tape layers: 3, 6, 9, and 12, (e) spindle speed: 2000, 2250, 2500 and 2750 rpm, (f) feed rate: 0.10, 0.11, 0.12, and 0.13 mm/rev. A Leadwell V-30 CNC machining centre with optional 4 axis rotary table was used to carry out the drilling operations in this investigation. The Scotch tape layers were placed only on the top side of CFRP. The experiments were designed in a way that one parameter may vary while other parameters were kept constant. Figure 1 shows the experimental set-up and photograph of drilled holes. A total of 16 holes were drilled at different experimental conditions, as listed in Table 1. The circularity and diameter errors were measured in coordinate measuring machine (CMM) Discovery II Model D-8. Each hole was measured in three different locations along the depth. Machined surface and delamination were examined under an Olympus SC100 optical microscope (Tokyo, Japan). The roughness of the machined surface was measured using a portable stylus-type surface profilometer (SJ-201; Mitutoyo Surftest, Washington, DC, USA). The average surface roughness (Ra) was measured using Mitutoyo surface roughness tester using cut-off length 0.08 mm with evaluation length 0.4 mm. The measurements were repeated 3 times to reduce the error. Machining force and torque were obtained by a Kistler 9124B rotating dynamometer while Dynoware 2825A (28 Channels) software was used to provide and evaluate high-performance and real-time graphics for cutting forces.

## 3. Results and Discussion

### 3.1. Thrust Force and Torque

**Figure 2:**
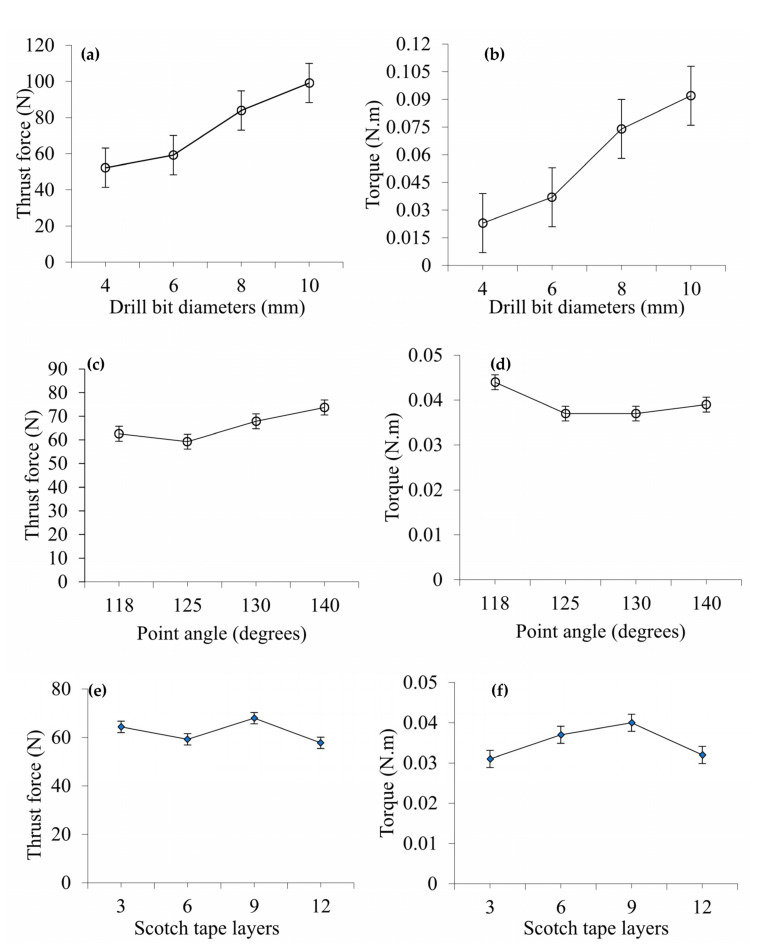
(**a**,**b**) Effect of drill bit diameters on thrust force and torque at point angle of 125°, 6 Scotch tape layers, spindle speed of 2250 rpm, and feed rate of 0.11 mm/rev, (**c**,**d**) Effect of point angle on thrust force and torque at drill bit diameter of 6 mm, 6 Scotch tape layers, spindle speed of 2250 rpm, and feed rate of 0.11 mm/rev, (**e**,**f**) Effect of Scotch tape layers on thrust force and torque at drill bit diameter of 6 mm, point angle of 125°, spindle speed of 2250 rpm, and feed rate of 0.11 mm/rev.

The effect of different drill bit diameters, varying point angles, and variable Scotch tape layers has been investigated on the resulting cutting forces (thrust and torque) during drilling. It shows the similar trends of increasing and decreasing of forces and torques during drilling holes with different drill bit diameters. The drilling forces and torques cannot become stable. The forces increase at the start of drilling at a high rate, then vary within a particular range before decreasing sharply towards the end of drilling. Similar results have been found in the previous research work [26]. Figure 2 shows the average value of thrust force and torques generated during the drilling process. Figure 2a,b presents the effect of different drill bit diameters on thrust force and torque, suggesting that force and torque increase with the increase of drill bit diameter and point angle. It can be seen that the observed output trend is almost linear. Average thrust forces obtained from drill bit diameters of 4, 6, 8, and 10 mm have been determined to be 52.24, 59.24, 83.92, and 99.12 N, respectively. Corresponding average torques obtained are 0.023, 0.037, 0.074, and 0.092 Nm, accordingly. This is mainly because as the drill bit diameters were increased from 4 to 10 mm, the respective area of contact between the drill bit periphery and CFRP sheet was also increased that resulted in an increased material removal rate (MRR). Therefore, while executing higher MRR from the CFRP sheet, the drill bit produced greater values of thrust and torque.

Figure 2c,d demonstrates the effect of point angle on average thrust force and torque where thrust force and torque do not change significantly. Initially both of these parameters decrease slightly with the initial increase of point angle and then increase slightly with a further increase in point angle. Maximum average thrust force obtained from point angles of 118°, 125°, 130°, and 140° have been found to be 62.58, 59.24, 67.85, and 73.71 N, respectively. In general, this shows that as point angles are increasing, the values of maximum average thrust force are also increasing accordingly. Maximum average torque obtained from point angles of 118°, 125°, 130°, and 140° have been determined to be 0.044, 0.037, 0.037, and 0.039 Nm, respectively. Point angle indicates the sharpness of drill bit tip at smaller point angles with a sharper tip. Point angle influences force and torque mostly at zones 1 and 2 and then point angle contributes to a small portion of the total force when the drill bit is completely engaged. A similar contribution of point angle also applies to the torque. Figure 2e,f shows the effect of Scotch tape layers on average thrust force and torque where these parameters are not altered significantly with the variation of Scotch tape layers. On average, both of these parameters initially increase as the number of Scotch tape layers increases and then decrease with a further increase in the number of Scotch tape layers. The average thrust force obtained from 3, 6, 9, and 12 Scotch tape layers are 64.37, 59.24, 67.98, and 57.78 N, respectively. Corresponding torques have been measured to be 0.031, 0.037, 0.04, and 0.032 Nm, accordingly. Thickness, hardness, and strength of Scotch tape are negligible when compared to that of CFRP plate. Therefore, the effect of the Scotch tape layer is very minor and the force and torque variations are not significant with the change of Scotch tape layer number.

**Figure 3:**
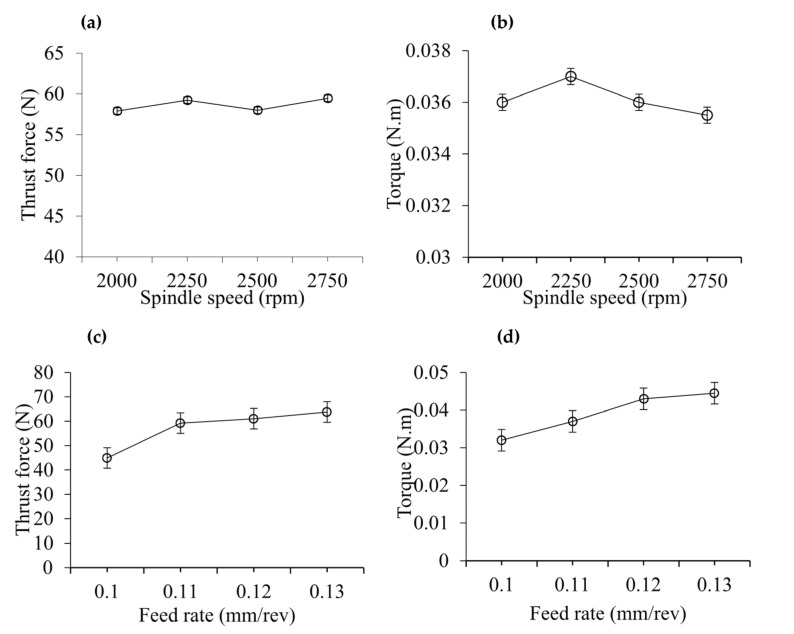
(**a**,**b**) Effect of spindle speed on thrust force and torque at drill bit diameter of 6 mm, point angle of 125°, 6 Scotch tape layers, and feed rate of 0.11 mm/rev, (**c**,**d**) Effect of spindle speed on thrust force and torque at drill bit diameter of 6 mm, point angle of 125°, 6 Scotch tape layers, and spindle speed of 2250 rpm.

Figure 3 illustrates average thrust force and torque recorded with different process parameters. It can be seen that thrust force and torque remain almost constant with the variation of spindle speed, as can be seen in Figure 3a,b. Average thrust force obtained from the spindle speeds of 2000, 2250, 2500, and 2750 rpm are 57.91, 59.24, 58, and 59.47 N, respectively. Their corresponding average torques are 0.036, 0.037, 0.036, and 0.0355 Nm, accordingly. The strength of CFRPs in the feed direction during drilling depends on the resin strength [20]. Machining speed affects force and torque in two ways by inducing strain rate and temperature depending on the composition of workpiece materials. Higher strain rate at higher speed increases the force and torque. On the other hand, thermal softening at higher speed reduces force and torque. The balance of these factors depends on workpiece materials. The material retained the hardening and softening effects due to that the force and torque remained almost constant with the variation of drilling speed. Figure 3c,d shows the effect of feed rate on average thrust force and torque. It can be seen that with the increase in feed rate, the average thrust force and torque was slightly increased. Average thrust force obtained from feed rates of 0.10, 0.11, 0.12, and 0.13 mm/rev have been found to be 44.91, 59.24, 61.04, and 63.79 N, respectively. For similar values of feed rate, corresponding torques are 0.032, 0.037, 0.043, and 0.0445 Nm, accordingly. The increase of feed rate increases material removal rate, resulting in an increase in force and torque.

### 3.2. Surface Roughness

**Figure 4:**
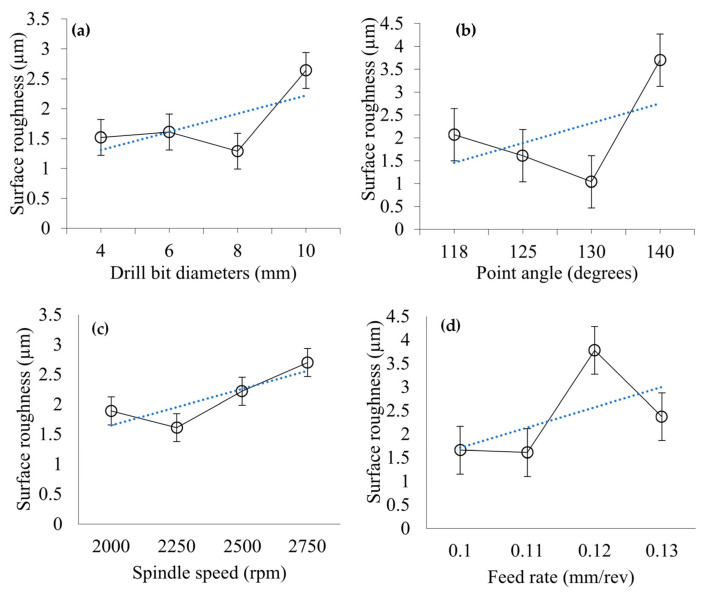
(**a**,**b**) Effect of drill bit diameter (at point angle 125°) and point angle (at drill bit diameter 6 mm) on surface roughness with 6 Scotch tape layers at the spindle speed of 2250 rpm, and feed rate of 0.11 mm/rev, (**c**,**d**) Effect of spindle speed (at feed rate 0.11 mm/rev) and feed rate (at spindle speed 2250 rpm) on surface roughness at drill bit diameter of 6 mm, point angle of 125° with 6 Scotch tape layers.

Figure 4 shows the effect of drilling process parameters on surface roughness of holes. Figure 4a,b shows the effect of drill bit diameter and point angle on the surface roughness of holes. It can be seen that surface roughness remains almost constant initially with increasing drill bit diameter. It further decreases and then increases again with increasing drill bit diameter. On the other hand, surface roughness decreases initially with increasing point angle, then it increases with a further increase in point angle. Surface roughness obtained from drill bit diameters of 4, 6, 8, and 10 mm are 1.52, 1.61, 1.29, and 2.64 µm, respectively. Surface roughness from point angles of 118°, 125°, 130°, and 140° are 2.07 µm, 1.61 µm, 1.04 µm, and 3.7 µm, accordingly. The behaviour of composite materials in the machining zone is very complex due to the presence of multiple materials with different fibre orientations, high strain rate, high strain, and high temperature. All these can contribute to the following: (i) inefficient load transfer between matrix-fibre and (ii) irregular failure behaviour of CFRPs to produce machining-induced surface defects [20]. Therefore, it is unlikely to have a specific trend of variation of surface roughness by changing drill bit diameter and point angle. However, the trendlines suggest that overall surface roughness is enhanced with increasing drill bit diameter and point angle. The fibres in CFRP are hard and require much higher temperatures to soften. On the other hand, the matrix material softens at lower temperatures. The linear speed at the periphery of the drill bit depends on its diameter where the speed at the periphery increases with the increase of tool diameter. During machining, higher temperature is generated at higher spindle speed (at larger diameter) which softens the matrix material and the bonding with harder fibre becomes flexible. The flexible bonding might induce pulling of the fibres rather than cutting. This generates a rougher machined surface at higher machining speed as well as higher tool diameter. The point angle represents the sharpness of the drill tip as well as the rate at which a hole is generated from piercing to full size. The bigger point angle provides a less sharp wedge of the trill tip and a higher rate of hole formation. These might have contributed to rougher surface for the drill bits with higher point angles.

**Figure 5:**
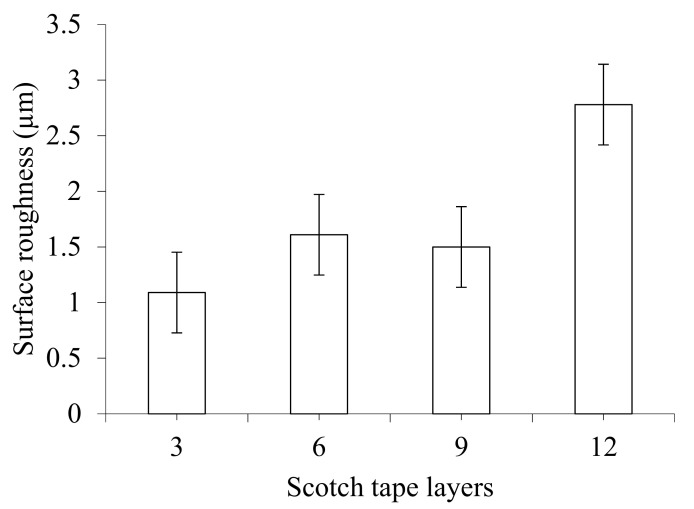
Effect of Scotch tape layer on surface roughness at drill bit diameter of 6 mm, point angle of 125°, spindle speed of 2250 rpm, and feed rate of 0.11 mm/rev.

Figure 4c,d shows the effect of spindle speed and feed rate on surface roughness. Surface roughness decreases slightly with an initial increase of speed; then it increases significantly with the further increase in spindle speed. On the other hand, surface roughness decreases at the beginning with increasing feed rate. Then it sharply increases and subsequently decreases with a further increase of the feed rate. Surface roughness obtained at spindle speeds of 2000, 2250, 2500, and 2750 rpm are 1.89, 1.61, 2.22, and 2.7 µm, respectively. Surface roughness determined at feed rates of 0.10, 0.11, 0.12, and 0.13 mm/rev are 1.66, 1.61, 3.78, and 2.37 µm, accordingly. Due to the irregular nature of surface generation after machining, specific trends of the variation of surface roughness with the change of speed and feed rate are not expected. However, the trendlines show that the roughness increases with increasing spindle speed and feed rate as increasing these two parameters promotes material removal rate and aggressiveness of drilling. The underlying mechanisms of co-relation between speed and surface roughness have already been discussed. It seems that the effect of the feed rate on the surface roughness follows a similar mechanism where the surface roughness increases with the increase of the feed rate. The higher feed rate generates higher temperature and softens the matrix material which contributes to the flexibility of fibres. The flexible fibres are difficult to cut but induce defects in the machined surface. Thus, the surface roughness increases with the increase of the feed rate. Figure 5 shows the effect of Scotch tape layer on surface roughness, which indicates that surface roughness increases at the beginning with increasing numbers of Scotch tape layers. Afterwards it remains almost constant and then the roughness increases sharply with a further increase in the Scotch tape layer number. Scotch tape layers on the top of machining surfaces may not directly contribute to surface roughness. The adhesive nature of Scotch tape layers may be able to seal any gap between workpiece and drill bit to prevent chip evacuation and damages to drilled surfaces. As the number of Scotch tape layers increases, the ability of the tape to resist chip evacuation improves, and thus it also applies to surface roughness.

### 3.3. Circularity

**Figure 6:**
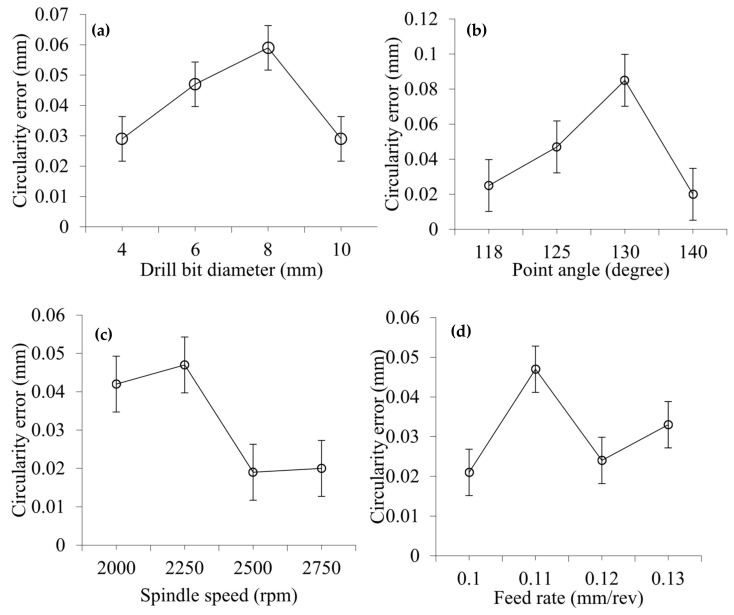
(**a**,**b**) Effect of drill bit diameter (at point angle 125°) and point angle (at drill bit diameter 6 mm) on circularity error with 6 Scotch tape layers at spindle speed of 2250 rpm, and feed rate of 0.11 mm/rev, (**c**,**d**) Effect of spindle speed (at feed rate 0.11 mm/rev) and feed rate (at spindle speed 2250 rpm) rate on circularity error at drill bit diameter of 6 mm, point angle of 125° with 6 Scotch tape layers.

**Figure 7:**
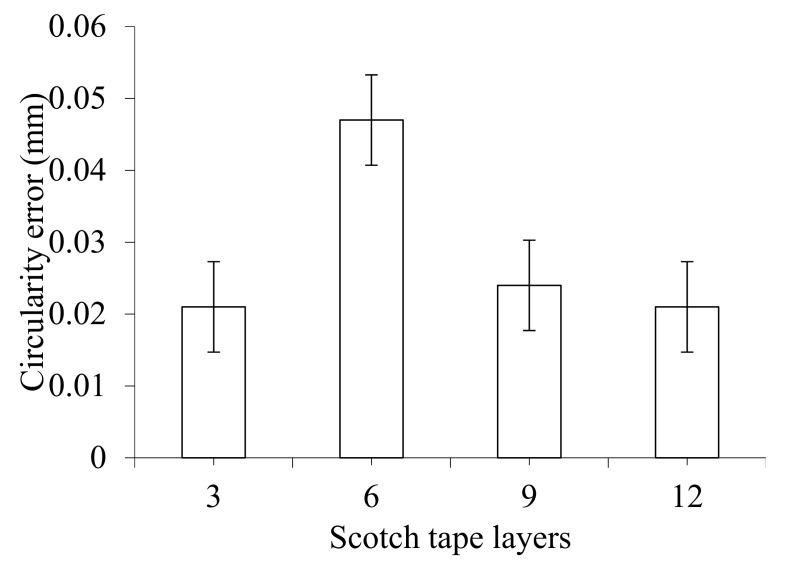
Effect of Scotch tape layer on circularity error at drill bit diameter of 6 mm, point angle of 125°, spindle speed of 2250 rpm, and feed rate of 0.11 mm/rev.

Figure 6 shows the effect of various drilling parameters on the circularity error. The effect of drill bit diameter and point angle on circularity error is presented in Figure 6a,b, respectively. It can be seen that the circularity error initially increased with drill bit diameter up to 8 mm but started decreasing with further increases in drill bit diameter. This indicates that the CFRP sheets are subjected to delamination and abrupt material removal fashion when operated using small sized drill bits. Circularity errors for drill bit diameters of 4, 6, 8, and 10 mm are 0.029, 0.047, 0.059, and 0.029 mm, respectively. A very similar trend of circularity has been noticed with the variation of point angle. Circularity errors obtained for point angles of 118°, 125°, 130°, and 140° become 0.025, 0.047, 0.085, and 0.020 mm, accordingly. The results showed that the pointed drill bit, with point angle 118°, produced a circularity error of 0.025 mm, indicating that the gradually evolved invading of the CFRP sheet is helpful. On the other hand, the drill bit with a point angle of 140° resulted in a circularity error of 0.020 mm, which might be because of higher thrust force (refer Figure 2c). The effect of spindle speed and feed rate on circularity error is illustrated in Figure 6c,d, respectively. From the graphs, it can be seen that the circularity error slightly increases initially with increasing drill bit diameter but decreases dramatically and then slightly increases with a further increase in drill bit diameter. A very similar trend with a higher increasing or decreasing rate of circularity has been observed with the variation of feed rate. Circularity at spindle speeds of 2000, 2250, 2500, and 2750 rpm is 0.042, 0.047, 0.019, and 0.02 mm, respectively. Circularity at feed rates of 0.10, 0.11, 0.12 and 0.13 mm/rev is 0.021, 0.047, 0.024 and 0.033 mm, respectively. The effect of Scotch tape layers on circularity error is depicted in Figure 7, which shows that circularity error increases at the beginning as the number of Scotch tape layers increases; then it declines at a different rate with a further increase in the number of Scotch tape layers. Circularity error for 3, 6, 9, and 12 Scotch tape layers are 0.021, 0.047, 0.024, and 0.021 mm, respectively.

### 3.4. Diameter Error

**Figure 8:**
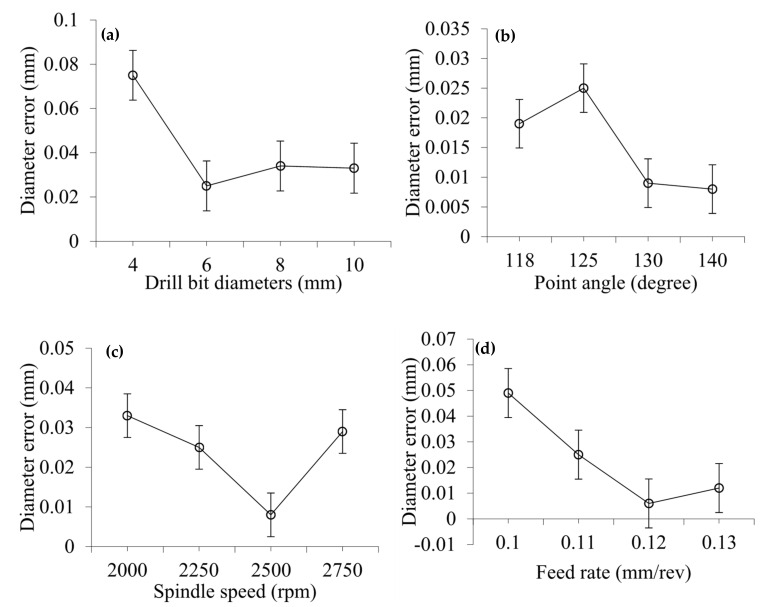
(**a**,**b**) Effect of drill bit diameter (at point angle 125°) and point angle (at drill bit diameter 6 mm) on diameter error with 6 Scotch tape layers at spindle speed of 2250 rpm, and feed rate of 0.11 mm/rev, (**c**,**d**) Effect of spindle speed (at feed rate 0.11 mm/rev), and feed rate (at spindle speed 2250 rpm) on diameter error at drill bit diameter of 6 mm, point angle of 125° with 6 Scotch tape layers.

Figure 8 shows the effect of drilling process parameters on the diameter error. The effect of drill bit diameter and point angle on diameter error is shown in Figure 8a,b. From the graphs, it can be seen that diameter error sharply decreases initially with the increase of drill bit diameter, but then increases slightly and remains almost constant with a further increase in drill bit diameter. On the other hand, diameter error increases initially with increasing point angle, but then decreases at different rates with a further increase of point angle. The diameter errors for drill bits of 4, 6, 8, and 10 mm are 0.075, 0.025, 0.034, and 0.033 mm, respectively. Diameter errors for point angles of 118°, 125°, 130°, and 140° are 0.019, 0.025, 0.009, and 0.008 mm, respectively.

**Figure 9:**
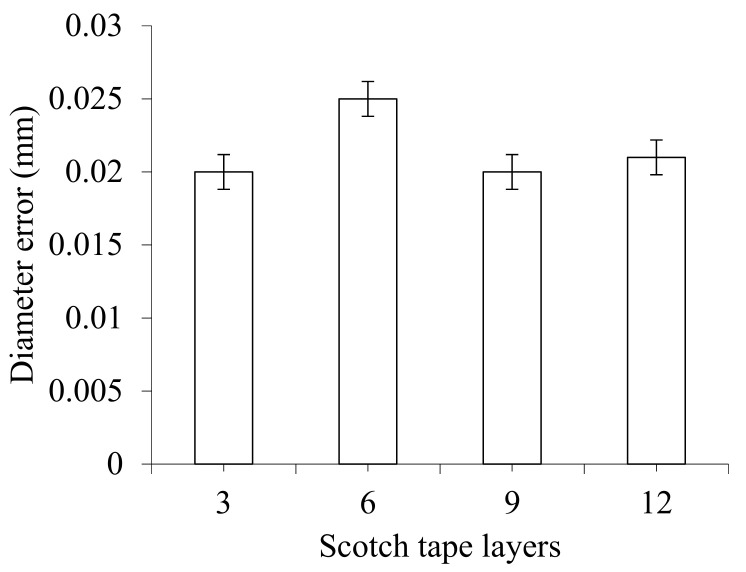
Effect of number of Scotch tape layer on diameter error at drill bit diameter of 6 mm, point angle of 125°, spindle speed of 2250 rpm, and feed rate of 0.11 mm/rev.

The effect of spindle speed and feed rate on diameter error is shown in Figure 8c,d. It can be seen that the diameter error sharply decreases at different rates with increasing drill bit diameter, but then increases drastically with a further increase of spindle speed. On the other hand, diameter error decreases dramatically at different rates with increasing point angle, but then increases slightly with a further increase of feed rate. Diameter errors at spindle speeds of 2000, 2250, 2500, and 2750 rpm are 0.033, 0.025, 0.008, and 0.029 mm, respectively. Diameter errors at feed rates of 0.10, 0.11, 0.12 and 0.13 mm/rev are 0.049, 0.025, 0.006 and 0.012 mm, respectively. The effect of number of Scotch tape layers on diameter error is shown in Figure 9. It is suggested that diameter error slightly increases with as the number of Scotch tape layers increases, but decreases slightly and then remains almost constant with a further increase in the number of Scotch tape layers. Diameter errors from 3, 6, 9, and 12 layers of Scotch tape are 0.02, 0.025, 0.02, and 0.021 mm, respectively.

### 3.5. Delamination

In general, delamination can be defined as the separation of layers within a composite laminated structure, which negatively affects their mechanical properties such as toughness, fatigue resistance, and hole quality. Delamination often occurs while machining this type of material. There are two types of delamination—peel up delamination and push down delamination. Peel up delamination happens at the entrance of a drilled hole. In this case, separated layers are affected by the peeling force applied to the drill bit. On the other hand, push down delamination happens at the exit of a drilled hole. Push down delamination is mainly affected by the reduced thickness of uncut layers within the composites themselves [5].

**Figure 10:**
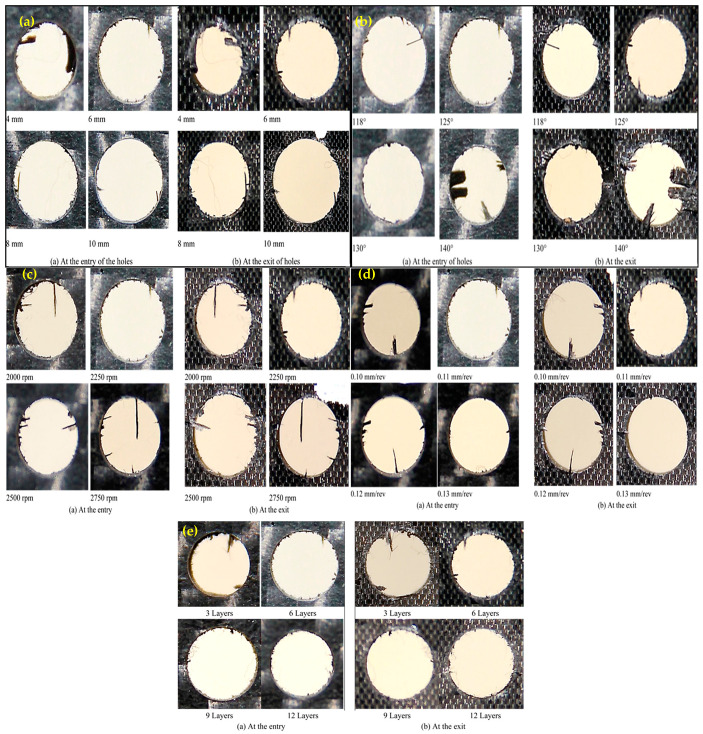
(**a**) Influence of drill bit diameter at the entry and exit of drilled holes (point angle of 125°, 6 Scotch tape layers, spindle Scheme 2250. Rpm, and feed rate of 0.11 mm/rev), (**b**) Influence of point angle at the entry and exit of drilled holes (drill bit diameter of 6 mm, 6 Scotch tape layers, spindle speed of 2250 rpm, and feed rate of 0.11 mm/rev), (**c**) Influence of spindle speed at the entry and exit of drilled holes (drill bit diameter of 6 mm, point angle of 125°, 6 Scotch tape layers, and feed rate of 0.11 mm/rev), (**d**) Influence of feed rate on the entry and exit of drilled holes (drill bit diameter of 6 mm, point angle of 125°, 6 Scotch tape layers, and spindle speed of 2250 rpm), (**e**) Influence of the number of Scotch tape layer at the entry and exit of drilled holes (drill bit diameter of 6 mm, point angle of 125°, spindle speed of 2250 rpm, and feed rate of 0.11 mm/rev).

**Figure 11:**
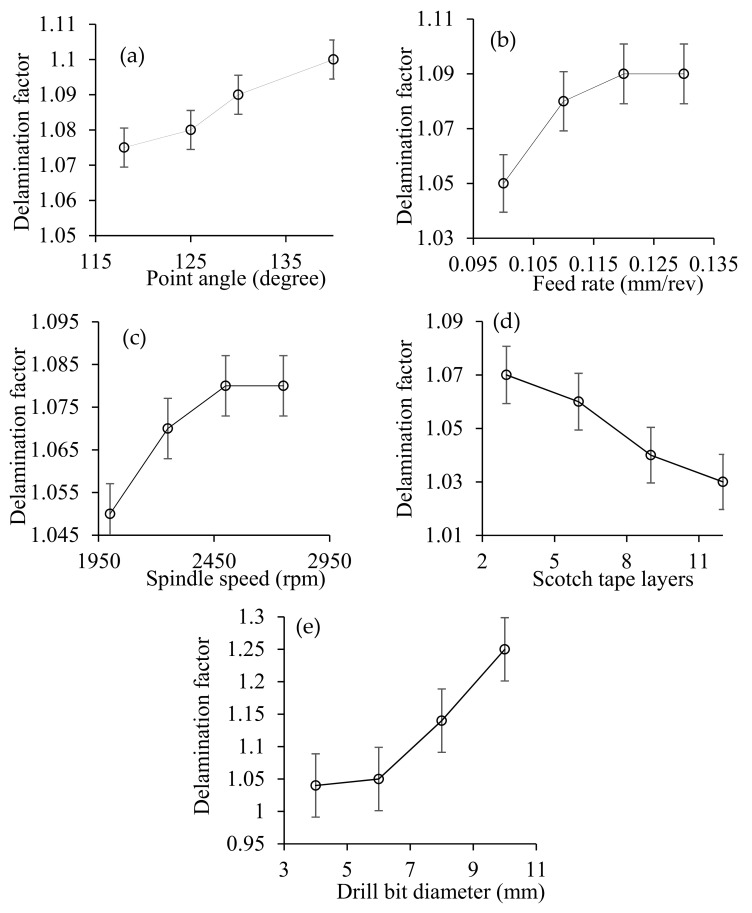
Influence of point angle (**a**), feed rate (**b**), spindle speed (**c**), Scotch tape layers (**d**), and drill bit diameter (**e**) on the delamination factor at the entry of the drilled holes for the similar conditions as presented in Figure 10.

**Figure 12:**
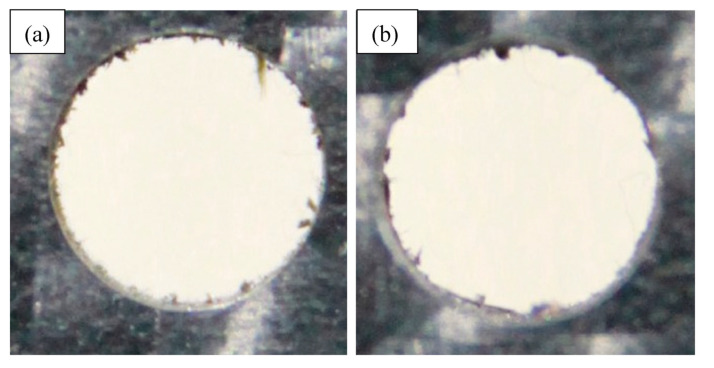
Drilled holes (**a**) with 3 layers of Scotch tape layer and (**b**) without Scotch tape layer.

Figure 10 shows a micrograph of drilled holes given various process parameters. The effect of the size of drill bits on the entry and exit of drilled holes are shown in Figure 10a. The delamination at the entry and exit of the holes is clearly seen. It is also evident that delamination at the exit is higher than that at the entry. The degree of delamination declines with larger drill bits at the exit and entry of the holes. Figure 10b demonstrates the impact of point angles on the entry and exit of delamination. It is clearly seen that less delamination has taken place at a lower point angle at the entry as well as at the exit of the holes. The lower point angle makes the drill bit pointed which helps it to gradually invade into CFRP sheet’ surface with a lesser thrust force. Being thermoplastic, the resin used in CFRP is rigid which reduces its ability to cope up with sudden thrust force. Here, the phenomenon of gradual invading using lower point angle is beneficial in controlling the delamination. The effects of spindle speed on the entry and exit of drilled holes are shown in Figure 10c. It is evident that spindle speed has negligible influence on delamination as the appearance of delamination is similar regardless of spindle speed. Figure 10d indicates that higher feed rate gives lower delamination at the entry as well as the exit of drilled holes. Figure 10e shows the effect of number of Scotch tape layers on the delamination at the entry and exit of drilled holes. As the Scotch tape layers are laid on top surface, the delamination is reduced significantly at the entry of the hole. In addition, increasing the number of Scotch tape layers reduces the delamination at the entry. The Scotch tape layer helps to hold the fibres attached to the matrix material and provides a support while the material is pushed out during drilling process. It seems that the higher number of Scotch tape layers increase the holding and supporting effects and reduces the delamination at the entry. This might have influenced the surface roughness and dimensional error. The delamination factors at the entry of the drilled holes were calculated as was the ratio of the maximum diameter of the delaminated zone to the diameter of the drilled holes. The variation of delamination factor is presented in Figure 11. The trends are very similar, as explained above. The entry of a hole without Scotch tape layers is presented in Figure 12 for comparison purposes which shows that worse delamination condition is comparable to entry with the Scotch tape layer.

## 4. Conclusions

The holistic analysis shows that that variation of input parameters such as drill bit diameter, point angle, Scotch tape layers, spindle speed, and feed rate significantly influence output parameters such as thrust force, torque, circularity, diameter error, surface roughness, and delamination on drilled holes. It has been identified that point angle has the most significant influence, followed by Scotch tape layers. Other input parameters such as drill bit diameter, spindle speed, and feed rate did not have a significant impact on the delamination. Increasing point angle, spindle speed, and feed rate causes an increase in delamination. However, increasing Scotch tape layers and drill bit diameter gives rise to a decrease in resulting delamination. Push down delamination is more likely to occur within the drilling of CFRPs as opposed to peel up delamination. Increasing drill bit diameter, point angle, and feed rate can induce an increase in output thrust force. Scotch tape layers and spindle speed have a weak relationship, with resulting maximum average thrust force as the output parameter. Increasing drill bit diameter, Scotch tape layers, spindle speed, and feed rate can cause an increase in torque as the output parameter. Increasing drill bit diameter and point angle yields an increase in resulting circularity. Conversely, increasing Scotch tape layers gives rise to a decrease in circularity. Spindle speed and feed rate have an insignificant relationship with circularity. Point angle, spindle speed, and feed rate have a strong relationship with resulting diameter error. Increasing point angle, spindle speed, and feed rate leads to a decrease in resulting diameter error. Increasing drill bit diameter, Scotch tape layers, and spindle speed induces an increase in surface roughness despite decreased surface roughness resulting from increasing point angles. The fluctuating data for surface roughness with increasing the feed rate implies their typically weak relationship as well.

## Figures and Tables

**Table 1:** Experimental Layout.

Experiment No.	Diameter (mm)	Point Angle (Degrees)	Scotch Tape Layers (No.)	Speed (rpm)	Feed (mm/rev)
1	4	125	6	2250	0.11
2	6	125	6	2250	0.11
3	8	125	6	2250	0.11
4	10	125	6	2250	0.11
5	6	118	6	2250	0.11
6	6	130	6	2250	0.11
7	6	140	6	2250	0.11
8	6	125	3	2250	0.11
9	6	125	9	2250	0.11
10	6	125	12	2250	0.11
11	6	125	6	2000	0.11
12	6	125	6	2500	0.11
13	6	125	6	2750	0.11
14	6	125	6	2250	0.10
15	6	125	6	2250	0.12
16	6	125	6	2250	0.13

## Data Availability

Not applicable.
